# Putative Involvement of Cytokine Modulation in the Development of Perioperative Neurocognitive Disorders

**DOI:** 10.2147/IJGM.S364954

**Published:** 2022-06-01

**Authors:** Christopher Pereira, Melanie Dani, Simon D Taylor-Robinson, Michael Fertleman

**Affiliations:** 1 Cutrale Perioperative and Ageing Group, Department of Bioengineering, Imperial College London, London, UK; 2 Department of Surgery and Cancer, Imperial College London, London, UK

**Keywords:** neuroinflammation, interleukins, central nervous system, perioperative neurocognitive disorders, blood-brain barrier, delirium

## Abstract

Following surgery, local cytokine-driven inflammation occurs, as part of the normal healing process. Cytokines in the central nervous system such as IL-6 and IL-8 may also be elevated. These cytokine changes likely contribute to neuroinflammation, but the complex mechanisms through which this occurs are incompletely understood. It may be that perioperative changes in pro- and anti-inflammatory cytokines have a role in the development of perioperative neurocognitive disorders (PND), such as post-operative delirium (POD). This review considers the current evidence regarding perioperative cytokine changes in the blood and cerebrospinal fluid (CSF), as well as considering the potential for cytokine-altering therapies to prevent and treat PND.

## Introduction

There is a growing appreciation that a peripheral insult to the body, such as surgery, can lead to cytokine-driven inflammation in the central nervous system (CNS).^1^,^2^ This may be a key factor in the development of perioperative neurocognitive disorders (PND), such as delirium.^3^ However, the mechanisms through which neuroinflammation occurs are currently unclear.^4^

## Neuroinflammation

Neuroinflammation is an inflammatory response within the brain or spinal cord that is partly mediated by cytokines.^5^ In the CNS, the predominant immune cells are the microglia, which are seen as the key driver of neuroinflammation, partly through release of intracerebral cytokines.^6^ Microglia have historically been considered to be either in the M1 (pro-inflammatory and injurious) or M2 (anti-inflammatory and protective) state.^7^,^8^ The M1 phenotype is activated by lipopolysaccharides (LPS) and the pro-inflammatory cytokine IFN-γ, leading to the production of oxidative metabolites, proteases and pro-inflammatory cytokines such as IL‐1β, IL‐6 and TNF-α.^9^,^10^ The M2 phenotype is activated by anti-inflammatory cytokines such as IL-4 and IL-13, and results in tissue repair and angiogenesis via arginase 1 and anti-inflammatory cytokines such as IL-10.^9^ While the M1 versus M2 phenotype is a useful classification, there is now an appreciation that the reality is likely to be more complex, with intermediate microglia states, rather than a clear binary role.^8^,^11^,^12^

The role of activated microglia is to guard against pathogens and tumour cells, so serving a key protective role in the brain.^9^ However, chronically activated microglia have been shown to release toxic factors such as cytokines and reactive oxygen species, which drive neuronal damage seen in many neurodegenerative diseases, such as Alzheimer’s disease.^13^ We hypothesise that this may also be a contributor to perioperative neurocognitive disorders.

## Perioperative Neurocognitive Disorders

PND is an umbrella term that includes cognitive impairment acutely after surgery (post-operative delirium), up to 30-days after surgery (delayed neurocognitive recovery) and up to 12-months after surgery (post-operative neurocognitive disorder).^14^ The commonest surgical complication in older people is post-operative delirium (POD), which affects around a quarter of patients admitted with hip fracture.^15^ The impact of delirium can be devastating and is distressing for patients, relatives and staff.^2^,^16^ Delirium is associated with an increase in mortality^17–19^ and poor functional recovery.^20^ Furthermore, delirium has been shown to be an independent risk factor for developing dementia.^21^ Understanding and treating PND is more important than ever with an ageing population.^22^,^23^

It is unknown whether PND arises from anaesthesia, surgery or both.^1^ Studies have shown that rates of PND are not affected by the type of anaesthesia used, possibly suggesting that the surgery, rather than the anaesthesia, drives PND.^14^,^24^ However, whether it is surgery or anaesthesia driving the process, there are currently no licensed drugs to prevent or treat PND, beyond trying to minimise the excessive use of sedation or analgesia.^4^ Cytokine-altering therapy offers a possible way of treating and preventing PND.

## Neuroinflammation in Neurodegeneration and PND

The role of neuroinflammation has been increasingly studied in neurodegenerative diseases, such as Alzheimer’s disease (AD),^25^ but less so in PND. Studies in AD show that the neuroinflammatory pathways involved in driving brain pathology are complex. Higher levels of the pro-inflammatory cytokine TNF-α are present in the CSF of patients with mild cognitive impairment who go on to develop AD.^26^ However, a degree of pro-inflammatory signalling may actually be beneficial in AD.^25^ Of note, studies using murine models of AD suggest that the pro-inflammatory cytokines, IL-6 and TNF-α, may accelerate amyloid beta plaque clearance.^27^,^28^

Animal models of PND suggest that like AD, neuroinflammation is present and that neuroinflammatory pathways may be fundamental in causing cerebral pathology. A study in mice showed that post-operative memory impairment was associated with increased levels of IL-1β in the hippocampus through microglial activation and that the memory impairment improved by functional inhibition of IL-1β.^29^

Although this review focuses purely on cytokines, this is only a piece of the neuroinflammatory puzzle. Other postulated mechanisms for the aetiology of PND include the role of the cholinergic anti-inflammatory pathway,^30^,^31^ oxidative stress,^1^ changes in neurotransmitters,^32^ lack of neurotrophic support and the microbiome-gut-brain axis.^31^ These ideas are not discussed here as they are not within the scope of this review.

## Cytokines

Approximately 200 different cytokines have been discovered to date.^33^ They are small proteins capable of diverse biological activities. CSF cytokines have been shown to be increased in depression,^34^ schizophrenia^35^ and neurodegenerative disorders such as AD.^25^ Some cytokines have pro-inflammatory effects, such as TNF-α, IL-1β, IL-6 and IL-8, and others, such as IL-4, IL-10 and IL-13, have anti-inflammatory effects.^36^ In most tissues, cytokines are released by macrophages, key components of the immune and inflammatory response.^37^ However, in the CNS, cytokines are mainly released from microglia, the predominant immune cell of the brain,^6^ as well as astrocytes.^38^ Microglia share similar pattern recognition receptors to macrophages and, once activated, create similar cytokine-mediated inflammatory responses.^3^ Cytokines in the CNS are considered to contribute to nearly all aspects of neuroinflammation.^1^

There are different mechanisms through which cytokines in the blood are thought to affect the CNS.^39^ These include entry at the BBB either because the BBB is weakened by ageing or neurodegeneration,^2^ or through the active transportation of certain cytokines, such as IL-1 and TNF-α, across the BBB.^40^ The endothelial cells that make up the BBB may also release cytokines.^39^ Furthermore, afferent fibres of the vagus nerve respond to cytokine increases in blood (such as IL-1β) to drive cytokine increases in the CNS.^41^ A final potential mechanism is that cytokines may diffuse between blood and CSF at the circumventricular organs (CVOs).^40^ CVOs are tiny specialised brain structures, located around the third and fourth ventricles. They link blood and CSF without a BBB, meaning they can be portals of entry into the brain.^42^

Investigating the association between plasma or serum cytokine changes and PND has not proved conclusive. Studies looking into the perioperative levels of serum IL-6 and POD have shown contrasting results. A study of 61 patients undergoing emergency surgery for hip fracture found that preoperative serum IL-6 levels were increased in patients with POD.^43^ These findings were replicated in a group of 23 patients undergoing complex aortic surgery.^44^ However, in a group of 151 patients having emergency surgery for hip fracture, serum IL-6 levels were not associated with developing POD.^45^ The authors speculated that these differing findings may be due to differences in cytokine analysis, sample timings, the composition of patient groups and the fact that prevalent and incident delirium groups were combined together. It may be that it is the balance between pro- and anti-inflammatory cytokines in blood, which triggers neuroinflammation, or that the cytokines involved are those which have not been studied. Alternatively, it may be that the neuroinflammatory response is driven by something other than blood cytokine increases. For example cortisol rapidly increases in blood following surgery.^46^ Cortisol can cross the BBB in its unbound form,^47^ and its levels have been shown to increase in the CSF following surgery.^48^

Analysis of cytokines in CSF, rather than in serum or plasma, appears more logical when investigating PND. Cerebrospinal fluid is in direct contact with the extracellular fluid of the brain and therefore reflects central biochemical changes more accurately than plasma or serum.^49^ Understanding the relationship between peripheral and central cytokines requires an understanding of the functioning of the blood–brain barrier (BBB), and how this may be disrupted at the time of surgery.

## Blood–Brain Barrier

The inflammatory theory of PND posits that some patients develop an accentuated systemic inflammatory response to surgery, whereby macrophages at the surgical site release excessive inflammatory mediators such as cytokines, driving CNS neuroinflammation.^2^ It was traditionally thought that the CNS was uniquely immunoprivileged, because the BBB created a physical barrier, preventing changes in the systemic circulation affecting the CNS. However, there is a growing appreciation that although the CNS is immunologically unique, it is not invulnerable to inflammatory changes in the systemic circulation.^50^ Most work looking into BBB dysfunction has taken place in neurodegenerative disorders, rather than in PND.^3^ In neurodegenerative disorders such as AD, BBB breakdown allows entry of blood-derived toxins into the brain. This can trigger the neurodegenerative process through multiple mechanisms, including the immune and inflammatory response, which drives neuronal injury, synaptic dysfunction, loss of neurons and brain connectivity.^51^

The BBB may become weakened in the perioperative period in patients without known neurodegenerative conditions.^52^ The accepted scientific methodology to investigate BBB permeability is the CSF/serum albumin ratio (Q-albumin).^53^ Albumin is made in the liver then secreted into the blood.^54^ As albumin is not made in the CSF, an increase in the CSF/serum albumin ratio is therefore suggestive of BBB breakdown.^55^ A study of 35 cognitively intact patients undergoing elective knee arthroplasty showed that the Q-albumin was significantly higher in the ten patients with more pronounced CSF cytokine elevation, suggesting that perioperative weakening of the BBB triggered a more pronounced CNS cytokine response.^48^ Furthermore, a study of 120 patients undergoing emergency surgery after hip fracture found that 14 patients had BBB dysfunction, all of whom also had POD. However, a further 55 patients developed POD despite an intact BBB, suggesting that BBB breakdown is not a prerequisite for developing POD, but may have a role to play in some cases.^56^ While these studies suggest perioperative BBB weakening may be an important driver of PND, they need to be replicated in larger studies.

## Preoperative Cytokine Levels

A number of studies in patients undergoing orthopaedic surgery have examined the preoperative serum or plasma and CSF levels of various cytokines, as summarised in Table 1. Studies looking into the differences between preoperative CSF IL-6 levels and POD have shown contrasting findings.^57^ A large study of 447 patients undergoing an elective hip or knee replacement found that preoperative IL-6 and TNF-α levels were significantly increased in patients with POD.^58^ However, a study of 61 patients undergoing emergency surgery for a broken hip found the opposite, with preoperative CSF IL-6 levels decreased in patients with POD.^43^ A further study of 151 patients who were also undergoing emergency surgery for a broken hip demonstrated that preoperative IL-6 levels in CSF were not associated with developing POD.^45^ The discrepancies in findings may be secondary to the fact that the POD subgroups within the trials were too small from which to draw valid conclusions. All of the above studies used the Confusion Assessment Method (CAM) to diagnose POD.^43^,^45^,^58^ However, the way in which the CAM was utilised differed. Lin et al using a single anaesthetist’s assessment, while Neerland et al using collateral information from nurses, relatives and hospital notes as well as a patient interview. This latter technique may have been more sensitive in picking up POD. A further confounder is that the study by Westhoff et al gave all participants haloperidol three times per day for 3 days from admission as delirium prophylaxis.

**Table 1:** Cytokine Changes in Cerebrospinal Fluid and Blood Before an Operation

Study	n =	Operation Type	Spinal or General Anaesthetic	Elective or Emergency Surgery	Plasma or Serum Sample Timings	Relevant Analysis	Major Cytokine Findings
Azim et al 2018^90^	98	Knee arthroplasty	Spinal	Elective	0, 4–6 and 72 hours after surgery	IL-6, TNF-α, leptin	Pre- and post-operative levels of IL-6, TNF-α and leptin do not predict post-operative pain levels.
Cape et al 2014^63^	43	Hip replacement or internal fixation	Spinal	Emergency	Before surgery	IL-1β, Il-1ra, IFN-γ, IGF-1	CSF Il-1ra and IL-1β were higher in patients with POD.
Chuang et al 2005^59^	20	Either open reduction and fixation for a broken hip or knee/hip arthroplasty	Spinal	Both	Before surgery	IL-1β, IL-2, IL-4, IL-6, IL-8, IL-10, IL-12p70, IFN-γ, TNF-α	IL-8 is increased in the CSF after peripheral trauma but is not present in the serum. Serum IL-6 levels increase after peripheral trauma, but not in the CSF.
Ji et al 2013^64^	83	Hip replacement	General and spinal	Elective	day 0 and day 7	IL-1c, IL-6	Patients with post-operative cognitive dysfunction had higher levels of IL-1β in their CSF before surgery.
Lin 2020^58^	447	Hip or knee replacement	Spinal	Elective	Before surgery, 24 hours after surgery	IL-6, TNF-α, cholinergic biomarkers	Levels of CSF IL-6 and TNF-α were elevated before surgery in patients with POD. Levels of plasma IL-6 and TNF-α were increased in plasma before and after surgery in patients with POD.
MacLullich et al 2011^62^	36	Hip replacement	Spinal	Emergency	Before surgery	IL-1β, IL-6, IL-8, IL-10, IL-12p70, TNF-α	Patients with POD had higher levels of CSF IL-8.
Neerland et al 2016^45^	151	Surgery for a broken hip	Spinal	Emergency	Before surgery	IL-6, sIL-6R, CRP	IL-6 levels in CSF and serum were not associated with POD.
Sajjad et al 2020^60^	133	Hip replacement	Spinal	Emergency	Not measured	IL-1β, IL-8, TNF-α	CSF IL-8 levels were higher in patients with POD and patients with depression.
Westhoff et al 2012^43^	61	Surgery for a broken hip	General and spinal	Emergency	Before surgery	42 Cytokines in CSF, IL-6 in serum	Pre-operative CSF levels of IL-1ra, IL-6 and Flt-3L were decreased in POD, while in the serum IL-6 levels were increased.

**Note**: A table summarising studies which have investigated cytokine changes in cerebrospinal fluid before an operation, with additional blood sampling.

**Abbreviations**: CSF, cerebrospinal fluid; Flt-3L, FMS-like tyrosine kinase 3 ligand; IFN-γ, interferon gamma; IGF-1, insulin-like growth factor 1; IL, interleukin; POD, Post-operative delirium; sIL-6R, soluble IL-6 receptor; TNF-α, tumour necrosis factor alpha.

A preoperative cytokine which has been consistently shown to be associated with POD in patients with hip fractures is that of IL-8. This was first found to be increased in the preoperative CSF of 11 patients undergoing emergency surgery for a broken hip.^59^ This finding has since been built upon, with a study in 36 patients showing CSF IL-8 levels in the preoperative period were associated with POD. This finding has more recently been replicated in a group of 133 patients.^60^

Studying certain cytokines, such as IL-1β, has been limited by the fact that some studies have been unable to detect it, because of very low levels before an operation.^60–62^ Studies which have been able to detect IL-1β suggest it may be associated with POD. A study in 43 patients undergoing surgery for a broken hip showed that levels of IL-1β were increased in patients with POD.^63^ The same finding was shown in a group of 83 patients undergoing elective total hip replacement.^64^ Other preoperative cytokines apart from IL-1β, which can be difficult to detect include IL-10, IL-12, IFN-γ and TNF-α.^60^,^62^,^63^ This severely limits the conclusions that can be drawn about these cytokines in the preoperative period.

## Post-Operative Cytokine Levels

While preoperative cytokine levels can be difficult to detect, it is possible to measure CSF cytokine levels in the post-operative period by leaving a spinal catheter in situ for 24 hours after an operation.^61^,^65^ Numerous cytokines have been shown to increase in the CSF after an operation, with greater increases in the cytokines IL-6 and IL-8 in the CSF than in serum, as summarised in Table 2. While these studies are informative, they have only been performed in patients undergoing cardiac, orthopaedic or vascular surgery, and the numbers involved are small. A study in 10 patients undergoing aortic valve replacement showed a 3.5-fold increase in CSF IL-6 (p<0.001) and a 12-fold increase in IL-8 (p<0.05) following surgery,^52^ as well as increases in CSF TNF-α levels, which were not seen in serum.^66^ A study of 35 patients undergoing knee arthroplasty found that cytokine elevation in the CNS was substantially greater than in serum, particularly in IL-8. However, this study did not measure IL-6.^48^ Finally, a recent study of 11 patients undergoing emergency surgery for a broken hip showed that 10 pro-inflammatory cytokine levels before the operation were typically low, with marked increases, particularly in IL-8, only after the operation. This suggests that a combination of fracture, pain, dehydration and a catabolic state do not drive neuroinflammation in the same way that surgery does.^65^

**Table 2:** Cytokine Changes in Cerebrospinal Fluid and Blood Before and After an Operation

Study	n =	Operation Type	Spinal or General Anaesthetic	Elective or Emergency Surgery	Intervention	Biofluid Sample Timings	Relevant Analysis	Major Cytokine Findings
Bromander et al 2012^48^	35	Knee arthroplasty	Spinal	Elective	Nil	0, 3 hours and the first morning after surgery	IL-1β, IL-2, IL-4, IL-5, IL-8, IL-10, IL-12p70, IL-13, IFN-γ, TNF-α	Post-operative cytokine increases in the CNS were greater than in serum, particularly in IL-8.
Buvanendran et al 2006^75^	30	Hip arthroplasty	Spinal	Elective	RCT with rofecoxib	0, 1, 3, 9, 24 and 30 hours after surgery	IL-1β, IL-6, IL-8, TNF-α	CSF levels of IL-6 increased after surgery, which was attenuated by preoperative rofecoxib.
Danielson et al 2018^79^	30	Open aortic valve surgery for aortic stenosis	General and spinal	Elective	RCT with methylprednisolone	24 hours before and 24 hours after surgery	IL-6, IL-8, TNF-α	Methylprednisolone before surgery decreased the levels of IL-6 and IL-8 in the serum and decreased IL-8 in the CSF.
Danielson et al 2020^67^	27	Knee or hip replacement	Spinal	Elective	Nil	0, 4, 8, 24, 32 and 48 hours after surgery	IL-6, IL-8, CXCL6, CCL3, CCL8	Higher CSF levels of IL-6, IL-8, CCL3, CCL8, and CXCL6 were found at 48 hours after surgery in patients with long-term cognitive decline.
Fertleman et al 2022^65^	11	Surgery for a broken hip	Spinal	Emergency	Nil	0, after surgery and day 1 after surgery	IL-1β, IL-2, IL-4, IL-6, IL-8, IL-10, IL-12p70, IL-13, IFN-γ, TNF-α	Cytokine rises were greater in CSF than plasma, with the largest increase in IL-8.
Hirsch et al 2016^61^	10	Major knee surgery	Spinal	Elective	Nil	0, 3, 6 and 18 hours after surgery	14 cytokines	Increases in pro-inflammatory cytokines were found in patients’ plasma and CSF after surgery. The changes in MCP, MIP-1α and MIP-1β were greater in CSF than serum.
Lindblom et al 2018^44^	23	Complex surgery of the thoracic aorta	General and spinal	Both	Nil	Before surgery, 8 am day 1 and 8 am day 2 after surgery	92 proteins with neurological relevance	IL-6 levels in CSF and serum are increased in post-operative spinal cord injury, IL-6 serum levels are increased in POD.
Nader et al 2001^78^	15	Lower limb revascularisation	Spinal	Unclear	Non-RCT with clonidine	0, post-operative samples taken but time points unclear	TNF-α	Clonidine decreases plasma and CSF levels of TNF-α after surgery.
Reinsfelt et al 2012^52^	10	Aortic valve bypass surgery	General and spinal	Elective	Nil	24 hours before surgery and 24 hours after surgery	Il-6, Il-8, S-100B	After aortic valve replacement there was a 3.5-fold increase in CSF IL-6 (p<0.001) and a 12-fold increase in IL-8 (p<0.05).
Reinsfelt et al 2013^66^	10	Aortic valve bypass surgery	General and spinal	Elective	Nil	24 hours before and 24 hours after surgery	IL-6, IL-8, TNF‐α	Using the cohort from Reinsfelt et al 2012^53^, showed that TNF-α levels increased in the CSF but not in the serum.
Renner et al 2011^76^	11	Hip arthroplasty	Spinal	Elective	RCT with etoricoxib	0, 1.5, 2, 4, 8, 12, 24, 26, 32 and 48 hours after surgery	IL-6, PGE2	Preoperative administration of etoricoxib lead to a reduction in serum IL-6 and pain levels, but no reduction was seen in CSF IL-6.
Vasunilashorn et al 2021^68^	29	Knee or hip total arthroplasty	Spinal	Elective	Nil	Before surgery and 1-month after surgery	IL-6, CHI3L1, CRP	In serum samples there was a significant increase in IL-6 at 1 month.
Wang et al 2013^91^	36	Aortic aneurysm surgery ± cardiopulmonary bypass	Spinal	Elective	Nil	Pre-incision, wound closure and every 4 hours for up to 5 days after surgery	IL-1α, IL-1β, IL-2, IL-4, IL-6, IL-10, IL-12p70, IFN-γ and TNF-α	Plasma IL-6 levels were higher in the patient group which underwent cardiopulmonary bypass.

**Note**: A table summarising studies which have investigated cytokine changes in cerebrospinal fluid and blood both before and after an operation.

**Abbreviations**: CCL, chemokine ligand; CHI3L1, chitinase 3-like protein; CNS, central nervous system; CSF, cerebrospinal fluid; CXCL, chemokine ligand; IFN-γ, interferon gamma; IL, interleukin; MCP, monocyte chemotactic protein; MIP, macrophage inflammatory protein; PGE, prostaglandin; POD, post-operative delirium; RCT, randomised controlled trial; S-100B, S100 calcium-binding protein B; TNF-α, tumour necrosis factor alpha.

Mechanisms by which post-operative cytokine changes may be associated with PND are less well studied, and again the available studies consist of small numbers of patients. In a group of 10 patients undergoing major knee surgery, the one patient who developed POD had a greater increase in CSF IL-6 and IL-8 than the patients without POD.^61^ In a group of 27 patients having either a knee or hip replacement, the six patients who went on to develop long-term cognitive decline had higher CSF cytokine levels of IL-6, IL-8, CCL3, CCL8, and CXCL6.^67^

The alternative to sampling from spinal catheters is to arrange a lumbar puncture (LP) when patients have recovered from surgery. Like the insertion of a spinal catheter, lumbar puncture is an invasive procedure with inherent risks, but it has been argued that it is under-utilised in investigating biomarkers of PND, as it is largely a safe procedure when undertaken by an experienced clinician.^32^ One recent large-scale study found that in 29 patients undergoing either a total knee or total hip replacement, IL-6 levels were increased in plasma, but not in CSF, after an LP was performed at 1 month following the operation.^68^ Levels of CRP were found to be increased in both CSF and plasma at 1 month. This may indicate that the cytokine-driven neuroinflammatory process had already resolved by 1 month after the operation.

## High Cytokine Responders

Some patients seem to have a more pronounced neuroinflammatory response than others. A study of 35 patients undergoing elective knee arthroplasty showed that 10 of the patients had particularly pronounced post-operative changes in nearly all the measured CSF cytokines (IL-2, 4, 5, 8, 10, 12p70, 13, TNF-α and INF-γ).^48^ In 11 patients undergoing hip fracture repair, 3 patients had similarly pronounced CSF cytokine responses following surgery.^65^ It remains to be seen whether those patients with a more pronounced CSF cytokine response are those that go on to develop PND. Of note, animal models suggest this may be the case, as in rats undergoing abdominal surgery, the magnitude of neuroinflammation was correlated with POD.^69^

## Cytokine Intercorrelation

It has consistently been shown that plasma or serum cytokines do not correlate with CSF cytokines in the post-operative period, with no association found between IL-6,^45^,^70^ IL-8^60^ and 10 pro-inflammatory cytokines.^65^ This is disappointing as the use of blood-based biomarkers is more attractive than CSF biomarkers due to the fact that obtaining CSF involves a more invasive process, which carries some risk.^71^

It is noteworthy that when multiple CSF cytokines are measured, they show strong intercorrelation.^48^,^65^ This suggests that multiple cytokines may be synergistically involved in driving neuroinflammation, but that this is limited to the CNS and may not be the case in the periphery.

## Cytokine Manipulation

While the particular role of CSF cytokine changes in PND needs to be determined, the changes described earlier do raise the possibility of prevention and treatment of neuroinflammation for PND.^48^,^72^ Blockade of the inflammatory cytokine TNF-α using biologic therapy, such as infliximab has revolutionised the treatment of rheumatoid arthritis^73^ and inflammatory bowel disease.^74^ This raises the question of whether new or existing cytokine blockade treatments could have a role in treating and preventing PND.

A further possibility is whether targeted medication can aim to restore or maintain the integrity of the BBB in the perioperative period, so preventing or attenuating microglia activation. However, no such treatments currently exist.^51^

Medications other than direct cytokine inhibitors may have some merit in the management of PND. Cyclooxygenase-2 inhibitors (COX-2i), used to treat inflammation in disorders such as rheumatoid arthritis, have shown different impacts on neuroinflammation. One study showed that the COX-2i, Rofecoxib, given before hip arthroplasty reduced post-operative CSF IL-6,^75^ but this was not replicated within a small cohort of 11 patients undergoing hip replacement receiving the COX-2i, Etoricoxib.^76^

Pain is a known risk factor for delirium.^16^ Patients often receive opiate medications to manage pain, but this is known to cause delirium, particularly in older patients.^77^ While opiates will likely remain the mainstay for managing pain for the foreseeable future, there may be a role for cytokine-altering medications. For example, preoperative administration of clonidine in patients undergoing vascular surgery has been shown to reduce peripheral and CSF TNF-α levels and postoperative pain scores.^78^

Perioperative corticosteroids have also been postulated to attenuate neuroinflammation in the post-operative period. In patients undergoing aortic valve replacement, preoperative administration of methylprednisolone caused a reduction in the levels of serum IL-6 and IL-8, a reduction in CSF IL-6, but an increase in IL-8 in the CSF. Rates of PND were not studied. The authors conclude that although the systemic inflammatory response was attenuated, the neuroinflammatory response was not.^79^ Large-scale studies in patients undergoing cardiac surgery have shown no reduction in POD following intraoperative methylprednisolone administration.^80^,^81^ However, a study in 140 patients undergoing non-cardiac surgery demonstrated that high-dose intravenous dexamethasone decreased the rates of PND in a randomised controlled trial. The authors demonstrated decreased levels of the neurotrophic cytokine, S100β, in post-operative serum of the steroid group, which the authors suggest may be a contributing factor to the decrease in PND.^82^ A major risk when using corticosteroids in the perioperative period is delayed wound healing, but some studies have shown that contemporaneous acute high-dose corticosteroid administration may not necessarily affect wound healing.^83^ Moreover, delirium is a known side effect of steroid administration.^84^ While steroids may eventually have a role in peri-operative cytokine reduction for PND, the fact they are a trigger for delirium suggests this is a complex mechanism, which is incompletely understood, and the risks that they may have must be carefully considered.

There are currently no registered trials or human data that use cytokine manipulating medication to prevent or treat PND.^1^,^72^ When developing medications to target PND, consideration must be given to the fact that blocking the inflammatory cascade may create an increased risk of infection due to immunosuppression.^3^ Furthermore, animal models of AD suggest that a degree of neuroinflammation may actually be beneficial.^25^ There may be a necessary amount of neuroinflammation in the body’s normal healing process that we do not yet fully understand. The future, therefore, may lie in a very targeted CNS approach to those with exaggerated cytokine responses. However, much remains to be understood about the role of cytokine changes in the role of PND before these measures can be applied clinically.

## Future Directions

A number of key criteria for future studies include the need for blinding, larger patient cohorts to enable subgroup analysis, standardised and detailed assessment of delirium, its possible aetiology and standardised collection and handling of CSF.^32^ In addition to this, cytokine analysis should not be limited to only a few cytokines per study. The brain is never exposed to a single cytokine, rather an array of both pro- and anti-inflammatory cytokines, and it may be the balance between these cytokine groups which proves to be crucial.^43^ The cytokine IL-8 has shown the most significant changes in CSF after an operation, and yet this has not been universally studied. The intercorrelation between different CSF cytokines in the post-operative period further demonstrates the need to test for a wide number of cytokines.^48^,^65^ Highly sensitive cytokine testing with electrochemiluminescence is readily available and straightforward to use with one assay offering ten pro-inflammatory cytokines in a single plate.^85^

Further considerations for future studies include the need for CSF samples to be taken in the post-operative period, as this is when the significant CNS cytokine elevations have been detected. Studies looking at serial CSF measurements after an operation need to be performed to enable detailed kinetics regarding the timings of cytokine changes to be understood. Consideration should be given to using patients undergoing thoracic vascular surgery, as this patient group frequently have a spinal catheter inserted as part of standard operative care.^86^ Future studies would need to consider the risks of removing excess levels of CSF, but the available studies have shown that the volume of CSF removed is not a risk factor for the commonest side effect from LP, a low CSF-pressure headache.^87^,^88^ It is noteworthy that the volume of CSF needed to perform cytokine analysis is typically low, with one assay only requiring 25μL of biofluid.^85^ Alongside the measurement of cytokines, analysis of the Q-albumin to understand perioperative changes in the BBB should also be undertaken.

A key, unresolved question is whether high-cytokine-responders are more likely to develop PND. If this hypothesis is proven to be correct, the potential to use cytokine-altering medications in this specific patient group is an attractive one. The INTUIT study is currently underway and is a large-scale observational study of 200 patients investigating PND. This includes the measurement of pro-inflammatory cytokines including IL-6 and IL-8 in the CSF and serum at 24 hours, 6 weeks and 1 year after surgery.^89^

## Conclusion

Cytokines have a fundamental role in driving neuroinflammation after an operation. However, our understanding of this process remains incomplete. It may be that a dysregulated or chronically activated neuroinflammatory process could be responsible for some elements of PND. By better understanding this process, the door may one day be open to targeted cytokine therapy to control excessive neuroinflammation and so prevent and treat PND.

## Abbreviations

BBB, blood–brain barrier; CNS, central nervous system; CSF, cerebrospinal fluid; IFN-γ, interferon gamma; IL, interleukin; PND, perioperative neurocognitive disorders; POD, post-operative delirium; TNF-α, tumour necrosis factor alpha.
